# Achieving Long Distance Sensing Using Semiconductor Laser with Optical Feedback by Operating at Switching Status

**DOI:** 10.3390/s22030963

**Published:** 2022-01-26

**Authors:** Bairun Nie, Yuxi Ruan, Yanguang Yu, Qinghua Guo, Can Fang, Jiangtao Xi, Jun Tong, Haiping Du

**Affiliations:** School of Electrical, Computer and Telecommunications Engineering, University of Wollongong, Northfields Avenue, Wollongong, NSW 2522, Australia; bn807@uowmail.edu.au (B.N.); yr776@uowmail.edu.au (Y.R.); qguo@uow.edu.au (Q.G.); canfang163@gmail.com (C.F.); jiangtao@uow.edu.au (J.X.); jtong@uow.edu.au (J.T.); hdu@uow.edu.au (H.D.)

**Keywords:** distance sensing, semiconductor laser, optical feedback, laser dynamics, Hopf-bifurcation

## Abstract

In this study, a novel distance sensing method is presented by using a semiconductor laser (SL) with optical feedback (OF) and operating the SL at a switching status happened between two nonlinear dynamic states (stable state and period-one state). In this case, without the need for any electronic or optical modulation devices, the laser intensity can be modulated in a square wave form due to the switching via utilizing the inherent SL dynamics. The periodicity in the switching enables us to develop a new approach for long-distance sensing compared to other SL with OF-based distance measurement systems and lift the relevant restrictions that existed in the systems. Moreover, the impact of system controllable parameters on the duty cycle of the square wave signals generated was investigated on how to maintain the proposed system robustly operating at the switching status. Both simulation and experiment verified the proposed sensing approach.

## 1. Introduction

A semiconductor laser (SL) with optical feedback (OF) has drawn much attention in recent decades due to its capability of generating rich dynamic states [1,2]. The attraction of an SL with OF (SLOF) for sensing and instrumentation is its merits of low cost in implementation, minimum part-count scheme, ease in optical alignment, and high dimensionality [3]. The external OF perturbation enables a stable locked SL to enter period-one dynamic through Hopf-bifurcation. For an SL in the stable state, the OF light is coupled with the light in its internal cavity; the coupled light modulates the frequency and intensity of the SL output light. This has led to the discovery of a class of laser interferometry, named self-mixing interferometry (SMI), also known as optical feedback interferometry [4]. Under certain external settings (e.g., increasing the OF strength), the system leaves the stable state, then period-one oscillation can be invoked through the Hopf-bifurcation and accompanied by an undamped relaxation oscillation [5]. The period-one oscillation produces an intensity-modulated optical wave and gives regular pulsation at microwave frequencies [6,7,8,9]. Period-one oscillation has been found in many significant applications, such as the generation of microwave/millimeter-waves [10], photonic microwave sensing/radar [11,12], radio-over-fiber systems [13,14], and other optical signal processing applications [15,16].

An interesting phenomenon called “switching” in an SL with external perturbance has drawn much attention for exploring its applications. In 2012, the work in [17] reported that the switching happened in the laser mutual coupled SLs and generated a laser intensity signal in square waveform with its time period equal to the time delay (or twice of the time delay). In 2017, the work in [18] reported that a tunable switching between the stable state and period-one state in an SL with OF can be achieved in a long-cavity region with about 10 m; it gives a potential application for square wave modulated photonic microwave generation. A very recent work [19] in 2021, demonstrated regular and irregular dynamics switching in a discrete-mode SL, also in a long-cavity region with about 32 m. The switching is found between stable state and period-one oscillation, as well as stable state and quasi-period/chaotic oscillation. Such switching status-induced square wave signals provide a new way for generating clock signals required by high-speed signal processing and communication systems. Various applications by making use of such switching-induced tunable square wave laser signals can be found, such as laser micromachining, and physical random number generations [20,21,22].

In this work, we propose to use an SLOF system operating at a switching status that happened between stable state and the period-one state for distance sensing. The system configuration of the proposed SLOF is similar to a conventional SMI that consists of an SL and external target. Regarding the SMI-based distance measurement, the work in [23] adopts a triangular modulated injection current to an SL by using an SL driver with frequency of 200 Hz and maximum depth of 0.01 mA (peak-to-peak). Within each triangular period, the fluctuation frequency of the laser power is related to the distance to be measured. This work achieved distance measurement with a range of 23 cm and resolution of 2.7 mm. The triangular modulation frequency and depth limit the measurement range and resolution. The work in [24] proposed to modulate the injection current in the form of sinusoidal waveform and, meanwhile, use an electro-optic crystal in the external cavity for the light phase modulation. A double-modulation technique was introduced to improve the measurement resolution. This work can measure up to 47.7 cm distance with 0.3 mm resolution. Another work reported in [25] achieved a 2 m range and 1.5 mm resolution, also by using a triangular modulated injection current with a frequency of 700 Hz and an amplitude of 1.5 mA. By using the pulse-counting method within each triangular period, the distance could be obtained. Modulating the SL with a stronger current can obviously increase the resolution. However, the modulation is limited by the electro-optical characteristic of SL. In a very recent work [26], an all-fiber laser SMI range finder with 15 m range and 47 mm relative error was reported. This work used a tunable fiber Fabry–Perot filter as a frequency selection device and requires applying a triangular modulated voltage signal onto the Fabry-Perot filter. The modulation frequency of 50 Hz and modulation voltage of 50 mV. This system has a wide sweep range of frequency and, thus, improved measurement range. Above reported SMI-based distance sensing systems require the use of an external electrical or optical modulation. The proposed approach using an SL with OF in this paper can remove those requirements. Besides, unlike above, SMI-based methods that require a feedback light from a target must located within half of the SL coherent length, the method presented in this paper can eliminate the coherent length restriction by operating an SL at switching status for distance sensing. Furthermore, the proposed sensing system can greatly improve the sensing performance in terms of range, and resolution compared to SMI-based sensing. In this paper, we also investigated the influence of the system controllable parameters on the duty cycle of the switching period and studied how to maintain the system robustly working at the proposed working status. Through utilizing the relationship developed between switching period and external cavity round trip time, the external target distance information could be detected.

Following this introduction, the SLOF system structure and its Hopf-bifurcation boundary between stable state and period-one state are presented in Section 2. Then, in Section 3, the generation of a square wave signal is demonstrated and the measurement algorithm for distance recovery is developed. In Section 4, experiments are conducted to verify the proposed distance sensing method. A conclusion is drawn in Section 5.

## 2. A Semiconductor Laser with Optical Feedback (SLOF) at a Switching Status

**Figure 1:**
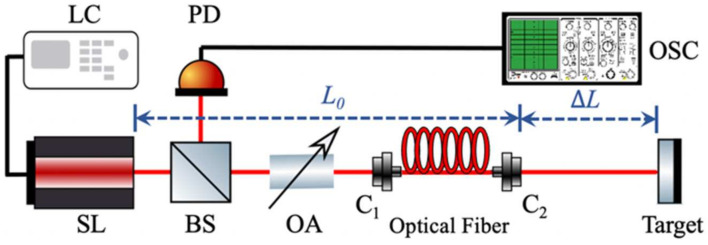
Schematic diagram of the SLOF sensing system. SL: semiconductor laser; LC: laser controller; BS: beam splitter; C: optical fiber coupler; PD: photodetector; OA: optical attenuator; OSC, oscilloscope. $L_{0}$: initial external cavity; ΔL: distance to be measured.

The schematic diagram of the proposed SLOF sensing system is shown in Figure 1. The emitted light from the SL passed through beam splitter (BS), optical attenuator (OA), and optical fiber, and then touched on an external target. Then, the light backscattered from the target and re-entered the SL. The SL was driven by a laser controller (LC) with an appropriate injection current and temperature. We used a piece of the mirror as the target to provide sufficient optical feedback to the SL. We defined external cavity length $L=L_{0}+\Delta L$, where $L_{0}$ is the initial external cavity length from the SL facet to the optical fiber coupler 2 ($C_{2}$), and ΔL is the distance to be measured from the coupler $C_{2}$ to the external target. The feedback strength of system could be adjusted by varying the OA. The BS was employed to direct a part of light into an external photodetector (PD); then, the microwave signal could be recorded by a high-speed digital oscilloscope (OSC). Comparing to the conventional SMI configuration, the main modifications were to replace the internal low speed PD with a fast speed external PD and to use a long optical fiber to form the external cavity.

The dynamic characteristics of SLOF is governed by the Lang and Kobayashi rate equations [27], which is a set of coupled nonlinear delayed differential equations, and can be numerically solved by the Runge–Kutta integration method.

(1) $$\frac{dE(t)}{dt}=\frac{1}{2}\left\{G_{N}[N(t)-N_{0}][1-\varepsilon \Gamma E^{2}(t)]-\frac{1}{\tau_{p}}\right\}E(t)+\frac{\kappa }{\tau_{in}}\cdot E(t-\tau )\cdot \cos\left[\omega_{0}\tau +\phi (t)-\phi (t-\tau )\right]$$

(2) $$\frac{d\phi (t)}{dt}=\frac{1}{2}\alpha \left\{G_{N}[N(t)-N_{0}][1-\varepsilon \Gamma E^{2}(t)]-\frac{1}{\tau_{p}}\right\}-\frac{\kappa }{\tau_{in}}\cdot \frac{E(t-\tau )}{E(t)}\cdot \sin\left[\omega_{0}\tau +\phi (t)-\phi (t-\tau )\right]$$

(3) $$\frac{dN(t)}{dt}=J-\frac{N(t)}{\tau_{s}}-G_{N}[N(t)-N_{0}][1-\varepsilon \Gamma E^{2}(t)]E^{2}(t)$$

In Equations (1)–(3), there are three variables, i.e., electric field amplitude E(t), electric field phase ϕ(t), and carrier density N(t). ϕ(t) is given by $\phi (t)=\omega (t)-\omega_{0}(t)$, where ω is the instantaneous optical angular frequency for an SL with optical feedback, $\omega_{0}$ is the unperturbed optical angular frequency for a solitary SL, $\omega_{0}=2\pi c/\lambda_{0}$ with $\lambda_{0}=780\;\mathrm{nm}$ is the laser wavelength, and c is the speed of light. $G_{N}=8.1\times 10^{-13}\;m^{3}s^{-1}$ is the modal gain coefficient, $N_{0}=1.1\times 10^{24}\;m^{-3}$ is the carrier density at transparency, $\varepsilon =2.5\times 10^{-23}\;m^{3}$ is the nonlinear gain compression coefficient, Γ=0.35 is the optical confinement factor, $\tau_{p}=2.0\times 10^{-12}\;s$ is the photon lifetime, $\tau_{s}=2.0\times 10^{-9}\;s$ is the carrier lifetime, $\tau_{in}=8.0\times 10^{-12}\;s$ is the internal cavity round-trip time. The controllable parameters include α the line-width enhancement factor, κ the feedback strength, J the injection current, and τ the external cavity round trip time, where τ=2L/c with L being the external cavity length.

**Figure 2:**
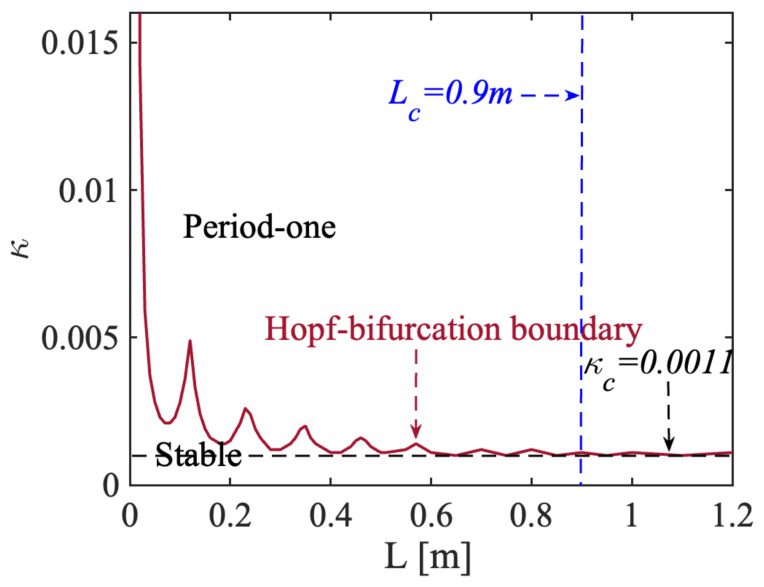
State diagram for an SLOF sensing system. Red line: Hopf-bifurcation boundary, where period-one state is above the boundary and stable state is below the boundary. Blue vertical dash line: critical external cavity length $L_{c}$. Black horizontal dash line: constant feedback strength $\kappa_{c}$.

The Lang and Kobayashi rate equations can be solved numerically by the fourth order Runge–Kutta integration method. We set the controllable parameters α=6 and $J=1.1J_{th}$, where $J_{th}$ the is threshold injection current. There was variance with the L from 0 m to 1.2 m with a step of 10 mm. For each L value, we gradually increased κ from 0 to 0.015 with a step of 0.0001, then observed the waveform of the signals I(t) (normalized $I(t)=[E^{2}(t)-\bar{E^{2}(t)}]/E_{0}^{2}$, where $E_{0}^{2}$ is the laser intensity without optical feedback), and recorded the corresponding dynamic state. Subsequently, a state diagram for the SLOF sensing system could be plotted, as shown in Figure 2. The Hopf-bifurcation boundary between the stable state and the period-one state was demonstrated in red color in Figure 2, indicating a fluctuation feature; when L was in a short-cavity region, the short-cavity region corresponded to the Hopf-bifurcation boundary with obvious fluctuation (e.g., L < 0.9 m). However, with the increase of L, the fluctuation of the Hopf-bifurcation boundary was gradually stabilized [12,28,29,30] and tended to a constant feedback strength (e.g., $\kappa_{c}=0.0011$ for current system parameter settings) when L was larger than the critical external cavity length denoted by $L_{c}$ (e.g., 0.9 m shown in Figure 2). We, hence, set the switching status for this SLOF with L larger than $L_{c}$, and κ near the Hopf-bifurcation boundary as the system operating condition. In this case, the laser intensity from the SLOF was modulated in a square waveform with its period linked to the external cavity length. In following simulations, we set the initial external cavity length $L_{0}$ as 1.5 m.

## 3. SLOF at Switching Status for Distance Sensing

**Figure 3:**
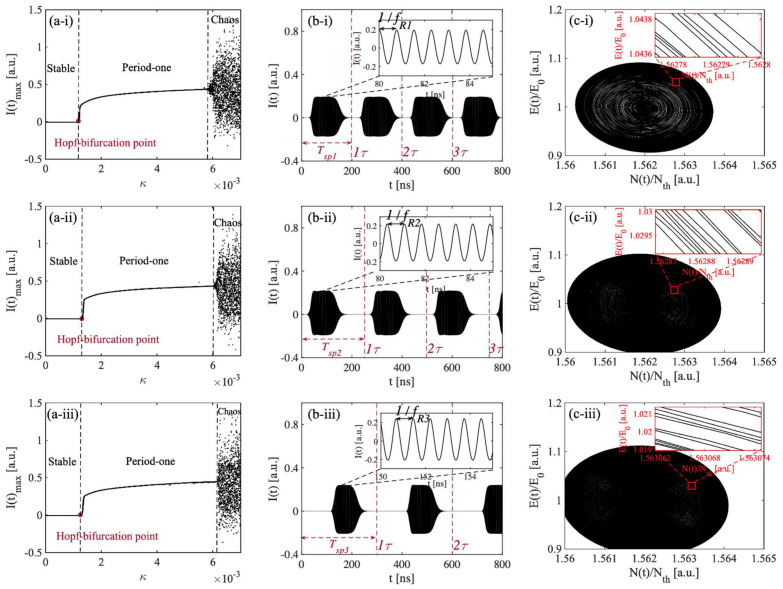
(**a**) Bifurcation diagrams. (**b**) Time-series signals I(t) near hopf-bifurcation point (that is the switching status). (**c**) Phase space images near Hopf-bifurcation point. (**i**) $\tau_{1}=200\;\mathrm{ns}$ (corresponding $L_{1}=30\;m$). (**ii**) $\tau_{2}=250\;\mathrm{ns}$ (corresponding $L_{2}=37.5\;m$). (**iii**) $\tau_{3}=300\;\mathrm{ns}$ (corresponding $L_{3}=45\;m$). The inset figures show the enlarged details.

To further examine the switching period feature between the stable state and the period-one state in a long-cavity region, we kept the initial external cavity length $L_{0}$ fixed at 1.5 m, α=6 and $J=1.1J_{th}$. The total external cavity length L was set at 30 m, 37.5 m, and 45 m, respectively, and their corresponding external cavity round trip times τ were 200 ns, 250 ns, and 300 ns, respectively. The relevant bifurcation diagrams were drawn and are shown in Figure 3a. As we were interested in the influence of the feedback strength κ on the SL states, we varied κ from 0 to 0.007 with a step size of 0.0001. At each κ, we obtained the corresponding maximum intensity $I{\left(t\right)}_{\max}$, and presented it on the bifurcation diagram in the ($I{\left(t\right)}_{\max},\kappa$) plane. Different states were indicated with their corresponding κ ranges. The bifurcation diagrams shown in Figure 3a clearly indicate the evolution route of the nonlinear dynamics in the SLOF system with an increasing κ, which moves from a stable state, period-one state, to chaos state. An undamped relaxation oscillation occurred after the Hopf-bifurcation point; in this case, the SLOF system could enter the period-one state. The laser intensity with a square wave-like envelope from the SLOF was observed and is shown in Figure 3b with $\kappa_{c}=0.0011$ near the Hopf-bifurcation boundary. It can be seen that the laser intensity signal showed a switching between the stable state and the period-one state. The relevant details of black areas can be read from the corresponding inset figures. In the period-one state section, the laser intensity exhibited a high frequency oscillation (denoted by $f_{R}$ for its peak frequency) shown in the inset of Figure 3b. Without the need for any high-speed electronic or optical modulation devices, microwave signals with square wave envelopes could be generated by utilizing the inherent dynamics of the SL with OF. Let us define the duty cycle as the time duration of period-one state over the square wave signal period in the time-series signal. The duty cycle shown in Figure 3(b-i)–(b-iii) are 75%, 63%, and 47%, respectively. These resultant square wave-like signals have potential applications to radio-over-fiber delivery of timing signals. Additionally, the SLOF system provides a possibility for the generation of a tunable square wave signal generation. In particular, the square wave signals shown in Figure 3b clearly indicates that the switching period $T_{sp}$ is equal to the external cavity round trip time τ. It can be better understood by using the phase space diagrams shown in Figure 3c. It can be seen that, in each phase space diagram, there are mickle concentric annular circles and that each phase space diagram is also a coexisting stable limit cycle attractor, which indicates that the amplitude of the period-one oscillation gradually increased during the transition of the time-series signal I(t) from the stable state to the period-one state and, furthermore, that the oscillation frequency $f_{R}$ remained unchanged. These features realize the regular conversion between two nonlinear dynamic states at the Hopf-bifurcation boundary.

**Figure 4:**
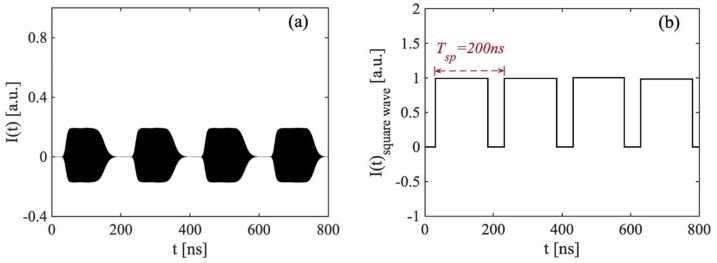
(**a**) Time-series signals I(t) obtained at the switching status with τ=200 ns and corresponding L=30 m. (**b**) Normarlized square wave signal $I{\left(t\right)}_{square-wave}$ obtained form I(t).

Based on the above simulation studies, an algorithm for utilizing the switching period $T_{sp}$ to measure distance was proposed. Taking the switching signal shown in Figure 3(b-i) as an example, the pre-set parameters were L=30 m, $L_{0}=1.5\;m$, and ΔL=28.5 m unchanged. The I(t) is shown in Figure 4a. We extracted its upper envelope; then, performing normalization and zero-crossing detection, the resultant square wave of I(t) is shown in Figure 4b. The switching period is the time between two raising edges and defined as $T_{sp}$. In this example, $T_{sp}$ is determined as 200 ns. Hence, we can obtain the corresponding recovered distance $\overset{\wedge }{L}$ is 30 m by utilizing the relationship of $\overset{\wedge }{L}=c\cdot T_{sp}/2$.

**Figure 5:**
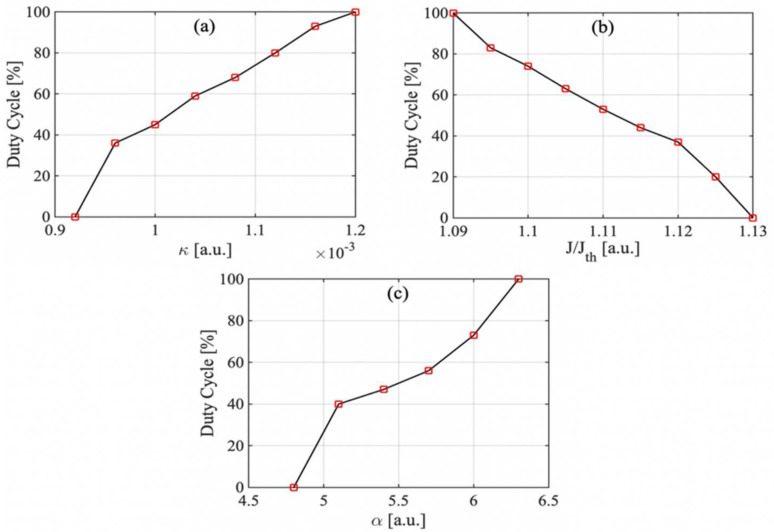
(**a**) Influence of κ on duty cycle with $J/J_{th}=1.1$, α=6, and L=30 m (τ=200 ns). (**b**) Influence of J on duty cycle with κ=0.0011, α=6, and L=30 m (τ=200 ns). (**c**) Influence of α on duty cycle with κ=0.0011, $J/J_{th}=1.1$, and L=30 m (τ=200 ns).

As for distance measurements using the proposed square wave generated in an SL with OF, it is critical to maintain the system to robustly operate at the switching status. Hence, it is important to study the impact of the system parameters (κ, J, and α) on the duty cycle and their characteristics. The influence of these parameters is shown in Figure 5a–c with a different varying system parameter, respectively. It can be seen that the duty cycle between 0% to 100% can be obtained for varying each system parameter within a certain range. Figure 5a shows an available κ from 0.00092 to 0.00120, corresponding to the duty cycle between 0% to 100%; the SLOF system can be used for distance sensing by measuring the square wave period. Outside this range, the SLOF system would fully enter into stable state or period-one state and the laser intensity signal will lose its periodicity; therefore, the square wave no longer existed in the system. In Figure 5b, starting from $J/J_{th}=1.09$ and gradually increasing injection current, the duty cycle tended to decline until it dropped to zero. This means that the SLOF system went from period-one state to stable state by crossing the Hopf-bifurcation boundary. Please note, in the SLOF system, the injection current J is a constant value; the adjustable duty cycle shown in Figure 5b illustrates the robustness of the SLOF system in terms of injection current and indicates that the SLOF system will not be affected by the current jitter. In Figure 5c, with the increase of α, the duty cycle of the square wave signal gradually increased from 0% to 100% within the α range of 4.8 to 6.3. For most SLs, their value of α is around 3~7 [30]; therefore, the switching presented in the proposed SLOF system is suitable for most SLs. In summary, with appropriate settings for these controllable parameters, it can be guaranteed that the switching phenomenon between stable and period-one state can occur, and that these parameters are able to control the duty cycle between 0~100%. These findings ensure the robustness of the proposed SLOF operating at the switching status for distance measurement.

## 4. Experiments

**Figure 6:**
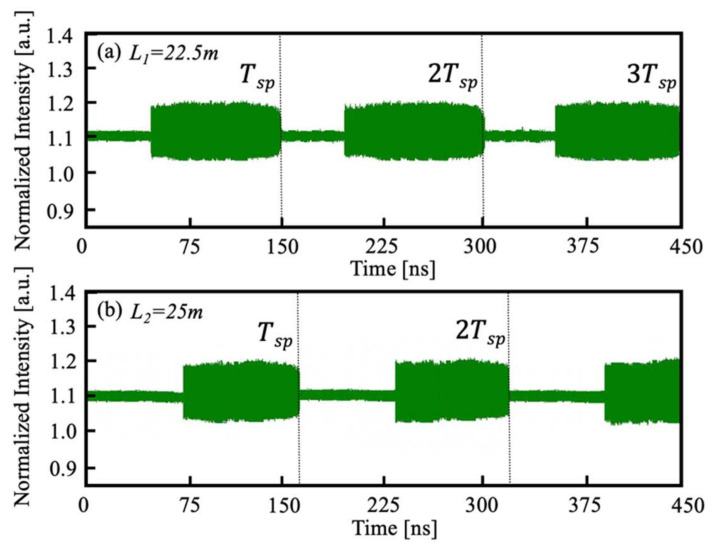
(**a**) Experimental square wave signal I(t) with a distance of $L_{1}=22.500\;m$. (**b**) Experimental square wave signal I(t) with $L_{2}=25.000\;m$.

We conducted a series of experiments and generated I(t) under different L to verify the proposed SLOF sensing system. An experimental setup is implemented according to the schematic diagram depicted in Figure 1. The laser source used in the experiments is a single-mode SL (Hitachi, Tokyo, Japan, HL8325) with a wavelength of 830nm. This SL is driven by the laser controller (Thorlabs, Newton, USA, ITC4001). The working temperature was T = 24.000 °C, the injection threshold current was $J_{th}=42\mathrm{mA}$, and the injection current was chosen at J=46.2mA. A small piece of mirror was used as the external target to provide sufficient optical feedback. The OA in Figure 1 is used to adjust optical feedback amount in the experiment. The light is split via a 50/50 BS and a direct part of light went into the external PD (Thorlabs, Newton, USA, PDA8GS). A high speed OSC (Tektronix, Beaverton, OR, USA, DSA70804) with a maximum sampling rate of 25GHz and analog bandwidth of 8GHz was used to observe square wave signals. As examples, we performed two different experiments to test our proposed SLOF system. In experiment 1, we denoted the total distance as $L_{1}=22.500\;m$, in experiment 2, we denoted the total distance as $L_{2}=25.000\;m$. For the convenience of the experiments, with the aid of fiber, we experimentally implement a long-distance sensing system that has the initial external cavity length $L_{0}=20.000\;m$. Then, the target was placed away from the fiber end with $\Delta L_{1}=2.500\;m$ and $\Delta L_{2}=5.000\;m$, respectively. The related experimental square wave signals can be observed and recorded by the OSC and shown in Figure 6a,b. From the experimental signals, we can determine the $T_{sp}$ are 150.04 ns and 166.68 ns, respectively. Therefore, by using the relationship of $\overset{\wedge }{L}=c\cdot T_{sp}/2$, the corresponding recovered $\overset{\wedge }{L}$ are 22.506 m and 25.002 m, which are both very close to the pre-set values 22.500 m and 25.000 m. We have repeated experiments for different distances under the same experimental operating conditions; the results agree with the pre-set values.

We discuss and analyze the measurement accuracy, uncertainty, and resolution of the proposed approach in the following. Regarding the measurement accuracy, the switching period $T_{sp}$ depends on the total optical length, which includes free space, fiber, BS, etc. However, in the proposed approach, there is no need to know the length of each part. We only need to set the SL operates at switching status by adjusting the feedback strength. The switching status can be recognized by observing laser intensity signals in the square waveform. Once switching status is achieved, from measuring $T_{sp}$, the total length which covered all parts can, thus, be obtained. Besides, as shown in Figure 5, as long as the SLOF system operates in the switching status between stable state and period-one state, the duty cycle is between 0% and 100% and the laser intensity signal will have its periodicity square wave character. Then, the proposed approach can be used to perform the distance measurement. Therefore, the change of the feedback strength by using an OA within a certain range will not affect the distance measurement results. Additionally, measurement uncertainty depends on whether the fixed-length section can be sufficiently immune from other disturbances, retain its original fixed value, and avoid the time measurement uncertainty. The oscilloscope we used in the experiments had time resolution ($T_{r}$) as 0.04 ns, which may cause uncertainty in obtained $T_{sp}$, it corresponds a resolution of distance measurement ($L_{r}$) as 6 mm by $L_{r}=c\cdot T_{r}/2$. The measurement resolution depends on the time resolution of the oscilloscope used in the experiment.

## 5. Conclusions

We propose a sensing system that realizes distance measurement by operating an SL with OF near the Hopf-bifurcation boundary to generate a switching between stable state and period-one state. To the best of our knowledge, this is the first work proposed to use the switching period for long-distance measurements. In this work, with the same system configuration as a conventional SMI system, but operating an SL at a different status, the proposed sensing system was able to achieve longer distance and relatively high resolution and to lift the restrictions in SMI that require electrical/optical modulation. In addition, the proposed sensing approach can achieve distance sensing beyond half coherent length. Furthermore, the influence of system controllable parameters on the duty cycle of the generated square wave sensing signals is also investigated. The result shows that each system controllable parameter has a wide available range. This has ensured that the proposed SL with OF system at switching can work robustly for long-distance sensing.

## Data Availability

The data presented in this study could be made available upon request to the corresponding author.
